# Certain dietary patterns including potatoes are associated with higher and lower diet quality and physiological measures in children and adults, NHANES 2001-2018

**DOI:** 10.3389/fnut.2022.987861

**Published:** 2022-11-10

**Authors:** Kristin Fulgoni, Victor L. Fulgoni

**Affiliations:** Nutrition Impact, LLC., Battle Creek, MI, United States

**Keywords:** NHANES, potatoes, Healthy Eating Index, dietary patterns, biomarkers

## Abstract

A large percentage of daily vegetable intake is attributed to white potatoes, but limited information is available on how potatoes are incorporated into dietary patterns in the US. Therefore, the objective of this study was to determine food patterns that include potatoes and to compare the associated diet quality and association with biomarkers to a food pattern without potatoes. Data from American subjects 2-18 and 19 years and older who participated in the What We Eat in America portion of the National Health and Nutrition Examination Survey cycles 2001-2018 were utilized in the current study. Diet quality was assessed using the Healthy Eating Index-2015. Anthropometric variables included body mass index (BMI), waist circumference, and weight. Biomarkers analyzed included glucose, insulin, triglycerides, HDL-, LDL-, and total cholesterol. Multiple food clusters containing potatoes were identified with several having higher and lower diet quality as compared to a food pattern without potatoes. Children and adolescents in one potato cluster had lower BMI, waist circumference, and body weight compared to those in a no potato dietary pattern, whereas adults in 3 potato clusters had higher anthropometric variables than those in a no potato pattern. In adults, some dietary patterns including potatoes were also associated with lower and higher HDL and total cholesterol and higher insulin levels. The percentage of calories from potatoes across patterns was small, ∼9-12%, suggesting the differences observed in diet quality and biomarkers were due to other food categories consumed in the pattern. This study suggests there are ways to incorporate potatoes as part of a healthy eating pattern but depends more on the other foods included in the diet.

## Introduction

Vegetables are an important component in a healthy dietary pattern and are associated with lower risk of all-cause mortality, cardiovascular disease, overweight and obesity, type 2 diabetes, deteriorating bone health, colorectal and breast cancers in adults (1, 2). The Dietary Guidelines for Americans (DGA) recommends between 1 and 2.5 cup equivalents of vegetables per day for children and adults, depending on total calories consumed (1). Potatoes are defined as a starchy vegetable in the DGA, of which 2-5 cups are recommended weekly (1). Potatoes contribute substantially to vegetable intake and are the top source of vegetables in children, representing 34% of vegetables, and one of the top sources in adults, contributing 25% to total vegetable intake (3).

Potatoes are a nutrient dense food that can be included as part of a healthy eating pattern due to high levels of several nutrients including potassium and dietary fiber, both defined as nutrients of public concern (1). Potassium intake has an inverse correlation with blood pressure, whereas higher fiber intake reduces the risk of coronary heart disease (1, 2). Depending on preparation, 100 g of potatoes can provide 535 mg and 2.2 g of potassium and fiber to the diet, respectively, representing 16-23 and 5.8-10.5% of the nutrient adequate intakes for children and adults (4–6). Previous studies have found potatoes contribute 4.0-6.7% and 3.8-6.9% of daily potassium and dietary fiber, respectively, in children and adolescents (7–9). For adults, potatoes have been reported as the 3rd and 4th highest sources of potassium and fiber, respectively, providing 6.7 and 6.4% of daily intake (10). In addition to potassium and fiber, potatoes contributed more than 5% daily intake of vitamins B6 and C, magnesium, copper, thiamin, niacin, and phosphorus while contributing less than 10 and 5% of daily energy and sodium intake, respectively (11, 12).

The association of potatoes with physiological measures and anthropometric variables is inconsistent based on previous studies and differ based on potato preparation. A recent review found positive and neutral associations between potatoes and weight gain, BMI, and waist circumference (13), but studies utilized different covariate sets and had moderate, serious, and critical risk of bias, possibly influencing the findings. Regarding physiological measures, a previous study found no associations between potato intake and blood pressure and BMI, but when focused on fried potato intake, associations were found in US women (14). Analyzing these physiological parameters association with dietary patterns including potatoes may give more clarity to the health impacts of potatoes when incorporated within a diet.

While numerous studies have reported on potato consumption and its impact on nutrient intake (11, 12), diet quality (15), and association with physiological variables (13, 14), there is a lack of information of dietary patterns with and without potatoes being associated with these variables. Therefore, the goal of the current study was to determine potato dietary patterns and how they differ from dietary patterns without potatoes and to assess the association of these patterns with biomarkers, body composition, and diet quality scores. It was hypothesized that dietary patterns including potatoes result in higher Healthy Eating Index 2015 (HEI-2015) scores overall as well as for total vegetables and can positively impact biomarkers and body composition measures.

## Materials and methods

### Study population and data source

The National Health and Nutrition Examination Survey (NHANES) is now a continuous survey representative of the non-institutionalized United States population that includes a dietary intake component, What We Eat in America (WWEIA). NHANES study designs and methods are available online (16, 17). WWEIA collects detailed information on the dietary intake of participating subjects divided into 15 main food groups, 46 subcategories, and over 150 unique categories. NHANES cycles 2001-2002, 2003-2004, 2005-2006, 2007-2008, 2009-2010, 2011-2012, 2013-2014, 2015-2016, and 2017-2018 were utilized in the current study. Male and female participants defined as either potato consumers or potato non-consumers 2 years and older (with subgroups 2-18 y and 19 + y) with complete 24-h recalls were analyzed in the current study. Pregnant and/or lactating subjects (*n* = 1,631), those with zero calories (*n* = 6) and subjects with unreliable dietary reports (*n* = 10,163), as determined by the USDA, were removed from the sample, resulting in a sample size of 72,584. For participants 12 years and younger, other individuals with the most knowledge of their dietary intake were used as proxies. WWEIA is collected using the Automated Multiple Pass Method (AMPM) (18) which is a five-step process with multiple passes which increases the accuracy of dietary reporting. NHANES protocols have been approved by the Research Ethic Review Board and individual personal information is not included in publicly available NHANES data.

### Dietary pattern analysis

Individual food intakes were determined using relevant Food Patterns Equivalent Databases (19). Potato consumers were defined as those with intake from WWEIA food categories potato chips (5002), white potatoes, baked or boiled (6802), French fries and other fried white potatoes (6804), and mashed potatoes and white potato mixtures (6806). Dietary patterns including potatoes and patterns without potatoes were determined using SAS 9.4 (SAS Institute, Cary, NC, USA). The SAS PROC CLUSTER command was utilized based on the percentage of calories from potatoes. The cluster analysis maximizes the differences in the percentage of calories coming from all food groups other than potatoes. Dietary patterns were defined as percentage of calories from potatoes and the 15 main food groups in WWEIA (20): dairy, protein foods, mixed dishes, refined grains, whole grains, snacks/sweets, fruit, vegetables, non-alcohol beverages, and alcoholic beverages. Cluster 0 was defined as the dietary pattern of potato non-consumers. After initial analysis where we examined 1, 2, 4, 6, 8, 10, 12, 14, and 16 clusters, we settled on further evaluation of six dietary clusters as a compromise between the percentage of variation explained as assessed by r-square, sample size of the clusters, and ease of interpretation. Children/adolescents and adults each had 3 unique clusters when compared to each other, therefore further analyses were conducted separately for these groups. The r-squared value for children and adults was of 0.418 and 0.383 in children and adults, respectively.

### Diet quality assessment and biomarkers

Diet quality was assessed using the HEI-2015 which measures the adherence to DGA recommendations. High scores are associated with higher intake of food components total vegetables, greens and beans, total fruit, whole fruit, whole grains, dairy, total protein foods, seafood and plant protein, and fatty acid ratio. Higher scores are also associated with lower intakes of foods recommended to be limited; sodium, refined grains, saturated fat, and added sugars. Components total fruits, whole fruits, total vegetables, greens and beans, total protein foods, and seafood and plant protein have a maximum of 5 points each, whereas whole grains, dairy, fatty acid ratio, refined grains, sodium, added sugars, and saturated fats have a maximum score of 10 points. Total scores were calculated as the summation of individual component scores and the maximum score is 100. Biomarkers and body measurements were collected during the Medical Examination Center portion of NHANES survey. Body weight, waist circumference, and BMI were collected with NHANES standardized protocols (16). BMI z-score for subjects 2-18 y was determined using CDC growth charts (21). Biomarkers were collected according to NHANES laboratory protocols and included HDL, LDL, and total cholesterol, fasting plasma glucose, fasting insulin, and fasting triglycerides.

### Statistical analyses

All analyses were adjusted for the complex sample design of NHANES. Regression analyses were used to determine differences in HEI-2015 total and component scores, body measures, and biomarker variables across dietary patterns. Covariates used in HEI-2015 analyses and body measures included age, gender, ethnicity, poverty income ratio, physical activity level, and current smoking status. For biomarkers, the above covariate set was used in addition to energy intake and BMI. Examination sample weights were used for intake analyses, anthropometric measures, HDL, and total cholesterol while fasting weights were used for fasting plasma glucose, fasting insulin, and fasting triglycerides. Statistical significance was set at <0.01.

## Results

### Cluster descriptions

In children, the six clusters identified had a percentage of calories from potatoes ranging from 8.6 and 11.7 percent (Table 1). Subjects in cluster 1 had the highest percentage of calories from protein foods (32.2%) and the lowest percentage from mixed dishes (7.0%). Those in cluster 2 had the highest percentage of calories from milk/dairy (31.1%) and the lowest percentage of calories from beverages (6.5%), while those in cluster 3 had the highest percentage of calories from potatoes (11.7%) as well as the highest percentages of calories from refined grains and beverages (16.3 and 13.8%, respectively). Subjects in cluster 4 had the highest percentage of calories from mixed dishes (51.8%) and the lowest percentages from milk/dairy, protein foods, refined grains and snacks/sweets (4.2, 3.5, 3.0, and 9.0%, respectively). Subjects in cluster 6 had the highest percentage of calories from snacks/sweets (44.5%) and the lowest percentage of calories from potatoes (8.6%).

**TABLE 1:** Potato dietary patterns of consumption (clusters) and mean percent calories (kcal) within food groups in American children and adolescents.

Cluster number	*n*	Potatoes	Dairy	Protein foods	Mixed dishes	Refined grains	Whole grains	Snacks/Sweets	Fruit	Vegetables	Beverages non-alcohol	Beverages alcoholic
0	15,965	0	12.42	11.57	24.35	11.62	3.32	18.64	3.17	1.01	10.07	0.14
1	2,090	11.24	7.81	32.24	7.03	8.70	2.12	12.56	2.33	1.07	11.29	0.04
2	971	8.77	31.07	9.31	9.31	8.41	3.40	16.45	3.08	0.86	6.52	0.08
3	3,399	11.73	11.50	13.48	7.92	16.34	3.10	13.10	2.76	1.18	13.77	0.03
4	1,082	10.26	4.24	3.48	51.83	2.99	1.52	8.98	0.99	0.45	13.59	0.04
5	3,496	11.11	8.75	9.10	30.45	7.44	2.14	14.19	2.34	0.78	10.02	0.45
6	1,035	8.58	6.60	10.02	8.34	5.88	1.74	44.47	1.56	0.99	8.55	0.01

Data from Day 1 recall from NHANES 2001-2018 for subjects 2-18 years of age (*n* = 28,038). Proc Cluster of SAS used to define clusters. WWEIA food groups were used to develop clusters based on % calories from each food group with potatoes as the centroid of the cluster. Cluster 0 represents no potato intake.

In adults, the percentage of potatoes in clusters 1-6 ranged from 8.6 to 12.2 percent of calories (Table 2). The adult clusters had a somewhat similar pattern as children and adolescents. Subjects in cluster 1 had the highest percentage of calories from potatoes (12.2%) as well as a high percentage of calories from refined grains and beverages (10.7 and 11.2%, respectively). Those in cluster 2 had the highest percentage of calories from mixed dishes (52%) and the lowest percentages from milk/dairy, protein foods, refined grains, and whole grains (2.5, 4.1, 3.2, and 1.3%, respectively). Subjects in cluster 3 had the highest percentage of calories from protein foods (43.9%) and the lowest percentage from snacks/sweets (5.6%) while those in cluster 6 had the highest percentage of calories from snacks/sweets (37.2%) and the lowest percentage of calories from potatoes (8.6%).

**TABLE 2:** Potato dietary patterns of consumption (clusters) and mean percent calories (kcal) within food groups in American adults.

Cluster number	*n*	Potatoes	Dairy	Protein foods	Mixed dishes	Refined grains	Whole grains	Snacks/Sweets	Fruit	Vegetables	Beverages non-alcohol	Beverages alcoholic
0	27,627	0	6.90	15.61	23.40	10.88	3.71	14.21	3.26	2.37	9.33	4.10
1	7,149	12.24	5.51	13.91	22.86	10.66	2.28	8.92	1.90	1.79	11.20	3.26
2	1,744	10.22	2.52	4.11	52.03	3.19	1.28	9.03	1.15	0.70	11.12	1.05
3	1,409	11.08	4.30	43.93	2.70	9.65	1.70	5.61	2.34	2.82	6.01	3.49
4	980	10.82	4.82	20.21	5.56	8.78	1.39	6.08	1.21	2.13	4.46	27.63
5	3,989	11.02	9.05	21.45	2.28	9.95	4.01	15.48	2.91	2.55	11.59	1.65
6	1,648	8.63	4.97	10.29	10.98	6.82	2.26	37.19	2.42	2.08	5.87	2.64

Data from Day 1 recall from NHANES 2001-2018 for subjects 19 years of age and older (*n* = 44,546). Proc Cluster of SAS used to define clusters. WWEIA food groups were used to develop clusters based on % calories from each food group with potatoes as the centroid of the cluster. Cluster 0 represents no potato intake.

### Diet quality measured by Healthy Eating Index 2015

In children, subjects in the no potato cluster had a HEI-2015 total score of 46.4 points (Table 3). Subjects in potato clusters 1, 2, and 3 had higher HEI-2015 total scores and those in clusters 4 and 6 had lower total scores compared to subjects in the no potato cluster. The highest HEI-2015 total score was observed in cluster 1 subjects at 50.5 points. The participants in this cluster had a higher score for components total vegetables, total protein foods, fatty acid ratio, and refined grains as well as lower scores for greens and beans, total fruit, whole fruit, whole grains, dairy, and sodium compared to those in the no potato cluster. Total protein foods, fatty acid ratio, and refined grains contributed 16, 30, and 38% to the difference in total scores, respectively. The lowest HEI-2015 total score was observed in cluster 4 subjects at 43.4 points. Subjects in this cluster had a higher score for total vegetables and fatty acid ratio and lower scores for greens and beans, total fruit, whole fruit, whole grains, dairy, seafood and plant protein, and sodium compared to those in cluster 0. Whole fruit, whole grains, and dairy contributed 16, 22, and 15% to the difference in total scores, respectively.

**TABLE 3:** Least square mean total and subcomponent Healthy Eating Index 2015 scores by potato cluster in American children and adolescents.

Variable	Cluster 0	Cluster 1	Cluster 2	Cluster 3	Cluster 4	Cluster 5	Cluster 6	*P* ^1^
								
	LSM (SE)	LSM (SE)	LSM (SE)	LSM (SE)	LSM (SE)	LSM (SE)	LSM (SE)	
Component 1 - Total vegetables	1.71 (0.02)	2.69* (0.05)	2.40* (0.07)	2.79* (0.04)	2.86* (0.06)	2.93* (0.04)	2.34* (0.06)	**<0.0001**
Component 2 - Greens and beans	0.97 (0.03)	0.71* (0.06)	0.73* (0.08)	0.64* (0.04)	0.67* (0.07)	0.74* (0.05)	0.66* (0.07)	**<0.0001**
Component 3 - Total fruit	2.59 (0.03)	2.29* (0.08)	2.22* (0.09)	2.63 (0.07)	1.93* (0.10)	2.36* (0.06)	1.80* (0.09)	**<0.0001**
Component 4 - Whole fruit	2.33 (0.04)	1.92* (0.08)	2.03* (0.11)	2.15 (0.07)	1.54* (0.11)	2.04* (0.06)	1.57* (0.10)	**<0.0001**
Component 5 - Whole grains	2.52 (0.05)	1.70* (0.10)	2.05* (0.13)	2.00* (0.08)	1.40* (0.12)	1.83* (0.08)	1.99* (0.11)	**<0.0001**
Component 6 – Dairy	7.21 (0.05)	5.25* (0.13)	9.24* (0.05)	6.65* (0.09)	6.45* (0.17)	6.55* (0.09)	5.09* (0.14)	**<0.0001**
Component 7 - Total protein foods	3.47 (0.02)	4.80* (0.02)	2.92* (0.07)	3.58 (0.04)	3.39 (0.09)	3.57 (0.04)	3.13* (0.07)	**<0.0001**
Component 8 - Seafood and plant protein	1.68 (0.03)	1.63 (0.09)	1.13* (0.09)	1.35* (0.06)	1.27* (0.10)	1.38* (0.06)	1.49 (0.10)	**<0.0001**
Component 9 - Fatty acid ratio	3.43 (0.04)	5.91* (0.14)	1.98* (0.14)	4.60* (0.10)	4.07* (0.18)	4.20* (0.10)	4.31* (0.16)	**<0.0001**
Component 10 – Sodium	4.87 (0.06)	4.41* (0.11)	6.24* (0.13)	5.38* (0.09)	4.38* (0.17)	4.67 (0.08)	6.80* (0.19)	**<0.0001**
Component 11 - Refined grain	4.48 (0.05)	7.62* (0.12)	7.68* (0.13)	6.32* (0.11)	4.40 (0.16)	5.54* (0.09)	5.91* (0.16)	**<0.0001**
Component 12 - Saturated fat	5.44 (0.05)	5.71 (0.13)	3.73* (0.16)	6.40* (0.09)	5.12 (0.17)	5.39 (0.09)	5.07 (0.16)	**<0.0001**
Component 13 - Added sugar	5.70 (0.05)	5.87 (0.10)	5.67 (0.15)	5.04* (0.11)	5.95 (0.20)	5.92 (0.10)	3.35* (0.19)	**<0.0001**
HEI-2015 total score	46.41 (0.21)	50.51* (0.46)	48.01* (0.53)	49.53* (0.35)	43.41* (0.50)	47.11 (0.36)	43.51* (0.43)	**<0.0001**

LSM, least square mean; SE, standard error; data represent U.S. individuals 2-18 years of age; genders combined; NHANES 2001-2018; *n* = 25,539; ^1^*P*-value denotes the testing of hypothesis of no overall cluster effect; *p-values derived from regression analyses testing the hypothesis of no difference between specific potato cluster and cluster 0 (no potatoes); covariates included age, gender, race/ethnicity, poverty income ratio level, physical activity level, and current smoking status. The bold values denote statistical significance (*p* < 0.01).

In adults, subjects in the no potato cluster had a HEI-2015 total score of 50.7 points (Table 4). Adults in potato clusters 3, 4, and 5 had higher HEI-2015 total scores and those in clusters 1, 2, and 6 had lower total scores compared to cluster 0. The highest total score was observed in cluster 4 subjects and was 4.1 points higher than cluster 0. Subjects in this cluster had higher scores for total vegetable, total protein foods, fatty acid ratio, sodium, refined grains, saturated fats, and added sugars and lower scores for greens and beans, total fruit, whole fruit, whole grains, dairy, and seafood and plant protein compared to cluster 0. Sodium, refined grains, saturated fat, and added sugars contributed 17, 32, 14, and 20% of the difference in total scores, respectively. The lowest total score was observed in cluster 2 subjects with 6.8 points less than cluster 0. Participants in this cluster had higher scores for total vegetables and lower scores for greens and beans, total fruit, whole fruit, whole grains, seafood and plant protein, sodium, refined grains, and saturated fat than cluster 0. Whole fruit, whole grains, and saturated fat contributed 12, 15, and 20% to the lower total scores, respectively.

**TABLE 4:** Least square mean total and subcomponent Healthy Eating Index (2015) scores by potato cluster in American adults.

Variable	Cluster 0	Cluster 1	Cluster 2	Cluster 3	Cluster 4	Cluster 5	Cluster 6	*P* ^1^
								
	LSM (SE)	LSM (SE)	LSM (SE)	LSM (SE)	LSM (SE)	LSM (SE)	LSM (SE)	
Component 1 - Total vegetables	2.78 (0.02)	3.62* (0.02)	3.54* (0.05)	3.59* (0.05)	3.45* (0.07)	3.48* (0.04)	3.19* (0.05)	**<0.0001**
Component 2 - Greens and beans	1.66 (0.03)	1.26* (0.04)	1.07* (0.07)	1.32* (0.08)	1.13* (0.08)	1.12* (0.05)	0.97* (0.06)	**<0.0001**
Component 3 - Total fruit	2.18 (0.02)	1.78* (0.04)	1.37* (0.07)	1.77* (0.09)	1.46* (0.07)	2.20 (0.05)	1.82* (0.07)	**<0.0001**
Component 4 - Whole fruit	2.16 (0.02)	1.74* (0.04)	1.28* (0.07)	1.75* (0.10)	1.41* (0.08)	2.11 (0.05)	1.97 (0.08)	**<0.0001**
Component 5 - Whole grains	2.63 (0.04)	1.84* (0.05)	1.46* (0.09)	1.40* (0.09)	1.43* (0.09)	2.62 (0.08)	2.09* (0.11)	**<0.0001**
Component 6 – Dairy	5.29 (0.04)	4.73* (0.06)	5.26 (0.10)	3.34* (0.11)	3.46* (0.14)	5.18 (0.08)	4.52* (0.10)	**<0.0001**
Component 7 - Total protein foods	4.14 (0.01)	4.23* (0.02)	4.15 (0.05)	4.97* (0.01)	4.31* (0.06)	4.38* (0.02)	3.83* (0.04)	**<0.0001**
Component 8 - Seafood and plant protein	2.44 (0.02)	1.97* (0.04)	1.70* (0.09)	2.66 (0.10)	1.95* (0.10)	2.08* (0.05)	1.89* (0.08)	**<0.0001**
Component 9 - Fatty acid ratio	4.82 (0.04)	5.33* (0.07)	4.54 (0.11)	6.44* (0.13)	5.63* (0.16)	4.91 (0.07)	4.69 (0.11)	**<0.0001**
Component 10 – Sodium	4.18 (0.03)	4.03 (0.06)	3.52* (0.12)	3.14* (0.15)	5.85* (0.17)	4.89* (0.09)	5.68* (0.12)	**<0.0001**
Component 11 - Refined grain	5.64 (0.04)	6.59* (0.06)	4.81* (0.13)	8.40* (0.10)	8.70* (0.10)	8.15* (0.06)	6.58* (0.11)	**<0.0001**
Component 12 - Saturated fat	6.10 (0.04)	5.93 (0.06)	4.61* (0.12)	5.10* (0.14)	7.46* (0.13)	5.66* (0.07)	5.13* (0.11)	**<0.0001**
Component 13 - Added sugar	6.65 (0.04)	6.37* (0.06)	6.53 (0.12)	8.24* (0.10)	8.55* (0.10)	5.51* (0.09)	4.70* (0.11)	**<0.0001**
HEI-2015 total score	50.67 (0.19)	49.42* (0.23)	43.85* (0.41)	52.12* (0.50)	54.80* (0.48)	52.29* (0.30)	47.08* (0.41)	**<0.0001**

LSM, least square mean; SE, standard error; data represent U.S. individuals 19 years of age and older; genders combined; NHANES 2001-2018; *n* = 40,911; ^1^*P*-value denotes the testing of hypothesis of no overall cluster effect; *p-values derived from regression analyses testing the hypothesis of no difference between specific potato cluster and cluster 0 (no potatoes); covariates included age, gender, race/ethnicity, poverty income ratio level, physical activity level, and current smoking status. The bold values denote statistical significance (*p* < 0.01).

### Potato clusters association with health biomarkers

In children, differences between subjects in cluster 0 and potato clusters were observed for BMI, waist circumference, weight, and total cholesterol (Table 5). Subjects in cluster 6 had 4, 2, and 5% lower BMI, waist circumference, and weight, respectively, compared to those in cluster 0. Participants in cluster 1 had 2% higher total cholesterol compared to cluster 0.

**TABLE 5:** Least square mean anthropometrics and biomarkers by potato cluster in American children and adolescents.

Variable	Cluster 0	Cluster 1	Cluster 2	Cluster 3	Cluster 4	Cluster 5	Cluster 6	*P* ^1^
								
	LSM (SE)	LSM (SE)	LSM (SE)	LSM (SE)	LSM (SE)	LSM (SE)	LSM (SE)	
BMI *z*-score (*N* = 17,065)	1.10 (0.01)	1.18 (0.03)	1.14 (0.03)	1.12 (0.02)	1.18 (0.04)	1.12 (0.02)	1.01 (0.04)	0.0169
Body mass index (kg/m^2^) (*N* = 25,146)	20.10 (0.06)	20.53 (0.18)	20.40 (0.16)	20.07 (0.12)	20.55 (0.28)	20.20 (0.11)	19.37* (0.17)	**<0.0001**
Waist circumference (cm) (*N* = 24,686)	69.02 (0.17)	70.12 (0.46)	69.18 (0.46)	68.87 (0.31)	70.64 (0.70)	69.23 (0.28)	67.30* (0.45)	**0.0003**
Weight (kg) (*N* = 25,290)	43.36 (0.18)	44.34 (0.53)	44.15 (0.46)	43.12 (0.34)	45.05 (0.85)	43.40 (0.34)	41.39* (0.45)	**0.0001**
Glucose, plasma (mg/dL) (*N* = 3,040)	95.17 (0.39)	94.22 (0.70)	94.13 (0.91)	93.83 (0.82)	94.61 (0.73)	95.47 (0.88)	96.51 (1.73)	0.5643
HDL-cholesterol (mg/dL) (*N* = 12,252)	50.66 (0.22)	51.71 (0.63)	49.24 (0.78)	50.95 (0.54)	50.39 (0.55)	50.90 (0.39)	51.20 (0.75)	0.3528
Insulin (uU/mL) (N=2,969)	15.41 (0.32)	15.49 (0.99)	16.08 (1.55)	15.27 (0.77)	15.44 (1.01)	15.42 (0.69)	16.36 (0.91)	0.9673
LDL-cholesterol (mg/dL) (*N* = 2,984)	90.82 (0.85)	93.18 (2.58)	101.4 (10.07)	85.90 (1.81)	87.74 (2.90)	88.70 (1.55)	98.97 (3.79)	0.0315
Total cholesterol (mg/dL) (*N* = 12,252)	161.0 (0.57)	164.7* (1.45)	162.5 (2.45)	160.8 (1.41)	159.2 (2.08)	160.7 (1.27)	162.8 (2.22)	0.1447
Triglyceride (mg/dL) (*N* = 2,993)	86.54 (1.73)	76.48 (3.94)	115.6 (15.25)	82.10 (4.15)	85.10 (6.16)	84.47 (2.81)	100.0 (20.81)	0.1301

LSM, least square mean; SE, standard error; data represent U.S. individuals 2-18 years of age; genders combined; NHANES 2001-2018; ^1^*P*-value denotes the testing of hypothesis of no overall cluster effect; *p-values derived from regression analyses testing the hypothesis of no difference between specific potato cluster and cluster 0 (no potatoes); covariates included age, gender, race/ethnicity, poverty income ratio level, physical activity level, and current smoking status for anthropometric variables; biomarkers included the above covariates plus energy and BMI. The bold values denote statistical significance (*p* < 0.01).

For adults, differences between subjects in cluster 0 and potato clusters were observed for BMI, waist circumference, weight, HDL cholesterol, insulin, and total cholesterol (Table 6). Subjects in cluster 2 had the largest differences in BMI, waist circumference, and weight at 5, 4, and 5% higher, respectively, compared to those in cluster 0. Participants in cluster 2 also had the lowest HDL cholesterol compared to those in cluster 0 with a difference of 3%. Cluster 4 subjects had the largest differences in HDL and total cholesterol at 13 and 5% higher, respectively, compared to those in cluster 0. Insulin was 7% higher in cluster 1 subjects, representing the largest difference in insulin compared to those in cluster 0.

**TABLE 6:** Least square mean anthropometrics and biomarkers by potato cluster in American adults.

Variable	Cluster 0	Cluster 1	Cluster 2	Cluster 3	Cluster 4	Cluster 5	Cluster 6	P^1^
								
	LSM (SE)	LSM (SE)	LSM (SE)	LSM (SE)	LSM (SE)	LSM (SE)	LSM (SE)	
Body mass index (kg/m^2^) (*N* = 40,254)	28.64 (0.08)	29.36* (0.14)	30.05* (0.27)	29.53* (0.30)	28.19 (0.23)	28.71 (0.16)	28.01 (0.24)	**<0.0001**
Waist circumference (cm) (*N* = 39,394)	97.98 (0.20)	99.97* (0.32)	101.6* (0.65)	99.92* (0.68)	97.68 (0.58)	98.22 (0.38)	96.68 (0.60)	**<0.0001**
Weight (kg) (*N* = 40,404)	81.79 (0.24)	84.26* (0.44)	85.94* (0.87)	84.64* (0.93)	81.07 (0.70)	82.24 (0.50)	80.45 (0.66)	**<0.0001**
Glucose, plasma (mg/dL) (*N* = 17,654)	104.6 (0.33)	106.5 (0.75)	106.8 (1.30)	108.1 (1.68)	102.0 (1.13)	104.8 (0.78)	103.9 (0.99)	**0.0058**
HDL-cholesterol (mg/dL) (*N* = 38,364)	53.18 (0.17)	52.56 (0.23)	51.78* (0.52)	53.49 (0.66)	60.10* (0.79)	52.08* (0.34)	52.90 (0.55)	**<0.0001**
Insulin (uU/mL) (N=17,315)	12.64 (0.14)	13.55* (0.31)	13.93 (0.48)	13.91 (0.90)	11.60 (0.60)	12.94 (0.33)	12.43 (0.40)	**0.0073**
LDL-cholesterol (mg/dL) (*N* = 17,141)	115.2 (0.49)	113.6 (0.91)	112.3 (1.72)	112.9 (2.01)	120.0 (2.15)	113.9 (1.04)	116.7 (1.85)	0.0751
Total cholesterol (mg/dL) (*N* = 38,365)	195.8 (0.46)	195.7 (0.70)	191.3* (1.41)	194.4 (2.01)	204.8* (2.13)	194.7 (1.09)	196.2 (1.48)	**<0.0001**
Triglyceride (mg/dL) (*N* = 17,480)	129.5 (1.58)	131.1 (2.49)	132.4 (6.05)	126.3 (6.48)	140.7 (6.45)	130.3 (2.98)	128.6 (7.21)	0.6216

LSM, least square mean; SE, standard error; data represent U.S. individuals 19 years of age and older; genders combined; NHANES 2001-2018; ^1^*P*-value denotes the testing of hypothesis of no overall cluster effect; *p-values derived from regression analyses testing the hypothesis of no difference between specific potato cluster and cluster 0 (no potatoes); covariates included age, gender, race/ethnicity, poverty income ratio level, physical activity level, and current smoking status for anthropometric variables; biomarkers included the above covariates plus energy and BMI. The bold values denote statistical significance (*p* < 0.01).

## Discussion

The current study objectives were to determine dietary patterns of potato consumers and non-consumers and their differences in diet quality, anthropometric variables, and biomarkers. This is the first study to analyze dietary patterns including potatoes to our knowledge. We defined six dietary patterns using a cluster analysis technique. In terms of diet quality, subjects in three potato clusters had higher HEI-2015 total scores compared to those with non-potato diets. Subjects in two and three potato clusters also had lower total scores in children and adults, respectively. Participants in the potato dietary patterns with the highest HEI-2015 total scores had higher component scores for refined grains, fatty acid ratio, and saturated fat. Subjects with the lowest total scores were mostly the result of low whole fruit and whole grains component scores. In children and adolescents, subjects in one potato cluster had lower BMI, waist circumference, and body weight compared to those in the no potato dietary pattern. On the other hand, in adults, subjects in three potato clusters had higher anthropometric variables compared to those in the no potato cluster. In adults, several differences in biomarkers were observed between participants in potato patterns and those in the non-potato pattern including both lower and higher HDL or total cholesterol levels and higher insulin levels in one cluster.

While total dietary quality scores were within 11 points of each other, patterns including differing levels of potatoes resulted in subjects having both higher and lower HEI-2015 total scores than those with dietary patterns without potatoes. Significant increases and decreases in component scores were present between dietary patterns with potatoes as compared to one without potatoes. These significant differences are small changes (approximately 11-32% of the maximum subcomponent scores) to component scores as compared to previous studies on individual foods such as oatmeal and apples (22, 23). The previous studies focused on foods that are more consistent with one or more specific subcomponents as compared to potatoes. The percentage of calories from potatoes across all clusters was within a small range (8.6 – 12.2%) in both children and adults suggesting the change in diet quality observed is attributable to foods other than potatoes. In the current study, the lowest HEI-2015 score was found for subjects in a potato cluster which had a middle range percentage of calories from potatoes and highest from mixed dishes in both children and adults. Subjects in clusters with high mixed dishes also were associated with higher body weight, waist circumference, and BMI in adults. Potatoes, especially fried potatoes, are commonly consumed as a side dish or incorporated into meals with high levels of refined grains and/or added sugars including sugar-sweetened beverages, burgers, meats, etc. (14). The most common mixed dishes during NHANES 2007-2018 included pasta excluding macaroni and cheese, rice mixed dishes, vegetable mixed dishes, and other Mexican mixed dishes which contain high levels of nutrients the DGA suggest to consume in moderation (1, 20). The food patterns observed maximized the differences in food categories other than potatoes and resulted in differing diet qualities. The DGA as well as previous dietary pattern studies (1, 24), in combination with these results suggest that typically one specific food or food category cannot be solely responsible for improved or decreased diet quality. The entirety of the meal/diet must be taken into account when assessing the impact of potato consumption on dietary quality and associations with body measures/biomarkers.

Similar to diet quality, the anthropometric differences observed in subjects with dietary patterns including potatoes compared to no potatoes may also be due to presence or absence of other foods consumed within the pattern and possibly due to preparation methods. In children and adolescents, one potato cluster was associated with lower BMI, waist circumference, and body weight compared to the no potato cluster. This dietary pattern included the lowest percentage of potatoes (8.58% of calories) and approximately 44% of calories from snacks and sweets. Findings on snack intake within children is controversial with studies reporting direct, inverse, and no relationships between snack intake and anthropometric variables depending on the type, amount, and energy density of the snack (25–28). The content of snacks is important to consider when determining their impact on body weight, BMI, and waist circumference. It’s possible the snacks consumed in this potato cluster were higher in protein, fiber, and/or whole grains which have been found to increase satiety, possibly resulting in less total energy consumption throughout the day (25). In regards to preparation methods, several reviews have reported both positive and inverse associations of potato intake with BMI, weight, and waist circumference (13, 29). More consistent findings are observed when looking specifically at French fry intake and adiposity, with higher intake being associated with weight gain and BMI, whereas findings for total or baked, boiled, and mashed potatoes and BMI are less consistent (13, 14, 30). The current study did not focus on the type of snack included in the dietary pattern nor preparation methods which may affect the associations found.

Differences were observed in biomarkers between subjects in potato clusters and those in the no potato dietary pattern. Interestingly, the highest HDL cholesterol was found in a cluster with subjects consuming 10.8% of calories from potatoes and 27.6% calories from alcoholic beverages. It is difficult to determine the food or food category that attributed this beneficial effect on cholesterol levels. Studies focused on the relationship between potato consumption and cholesterol are scarce with a recent review identifying two studies that found no difference in total, HDL, and LDL cholesterol between high and low potato consumption (29). Another source of increased HDL cholesterol may be the high percentage of alcohol intake in the cluster, although the percentage of alcohol in this dietary pattern is well above the recommended limits (1). Alcohol potentially has a favorable impact on HDL-cholesterol, but is dependent on the amount consumed as well as the type of alcohol (31, 32). In addition to differences in HDL cholesterol, subjects in one potato cluster had higher insulin levels than those in the no potato cluster. Participants in this dietary pattern had the highest percentage of calories from potatoes as well as the second highest percentage of calories from refined grains as well as non-alcoholic beverages. Both non-alcoholic beverages, which are typically sugar-sweetened beverages that contribute high levels of added sugars (33), and refined grains have been associated with higher insulin in previous studies (34). Similar to diet quality, the totality of the diet must be taken into consideration when assessing specific food and food categories relationships with biomarkers and cannot be attributed to potatoes alone.

A strength of the current study includes the use of a large sample size representative of the US population from NHANES separated into two age groups. A second strength was the utilization of a previously used technique to understand dietary patterns through cluster analysis (24). The current study has several limitations. NHANES depends on self-reported dietary intake which are subject to misreporting (35). Proxies were used for children under 12 who may have limited information on foods consumed outside the house, further adding to misreporting. Although covariates commonly used in intake studies were used, residual confounding may still be present. As with all observational studies, cause and effect could not be determined. Finally, the analyses focused solely on dietary patterns with and without potatoes so other foods within the diet likely have influenced the current results.

The current study identified several dietary patterns in children and adults that included potato consumption with varying levels of other food categories. Diet quality differed between subjects with food patterns including various levels of potatoes with clusters showing both improved and decreased diet quality as compared to food patterns without potatoes even though the percentage of calories from potatoes in all potato clusters was within a finite range (from 8.6-12% in both children and adults). Similar results were observed with biomarkers. Participants in several potato food patterns had decreased anthropometric variables in children compared to those with food patterns excluding potatoes. In adults, differences were observed in both anthropometric and physiological variables including HDL-, total cholesterol, and insulin. Given the finite range of calories from potatoes in clusters, these results suggest the differences in diet quality and biomarkers observed in the current study are likely attributed to the presence or absence of other food categories in the dietary patterns. These data also suggest potatoes can be a part of healthy dietary patterns when balanced with other nutrient-rich foods consistent with dietary recommendations.

## Data availability statement

Publicly available datasets were analyzed in this study. This data can be found here: National Health and Nutrition Examination Survey 2001-2018, https://wwwn.cdc.gov/nchs/nhanes.

## Ethics statement

The NHANES study procedures were reviewed and approved by the NCHS Ethics Review Board (for more details on specific protocols see https://www.cdc.gov/nchs/nhanes/irba98.htm). Written informed consent was obtained from all participants for their participation in this study.

## Author contributions

VF: conceptualization, methodology, and analyses. KF: writing – original draft preparation. VF and KF: writing – review and editing. Both authors read and agreed to the published version of the manuscript.
